# Author Correction: The influence of human genetic variation on Epstein–Barr virus sequence diversity

**DOI:** 10.1038/s41598-021-87274-z

**Published:** 2021-04-20

**Authors:** Sina Rüeger, Christian Hammer, Alexis Loetscher, Paul J. McLaren, Dylan Lawless, Olivier Naret, Nina Khanna, Enos Bernasconi, Matthias Cavassini, Huldrych F. Günthard, Christian R. Kahlert, Andri Rauch, Daniel P. Depledge, Sofia Morfopoulou, Judith Breuer, Evgeny Zdobnov, Jacques Fellay, the Swiss HIV Cohort Study, Karoline Aebi-Popp, Alexia Anagnostopoulos, Manuel Battegay, Enos Bernasconi, Jürg Böni, Dominique Braun, Heiner Bucher, Alexandra Calmy, Matthias Cavassini, Angela Ciuffi, Guenter Dollenmaier, Matthias Egger, Luigia Elzi, Jan Fehr, Jacques Fellay, Hansjakob Furrer, Christoph Fux, Huldrych F. Günthard, David Haerry, Barbara Hasse, Hans Hirsch, Matthias Hoffmann, Irene Hösli, Michael Huber, Christian R. Kahlert, Laurent Kaiser, Olivia Keiser, Thomas Klimkait, Lisa Kottanattu, Roger Kouyos, Helen Kovari, Bruno Ledergerber, Gladys Martinetti, Begoña Martinez de Tejada, Catia Marzolini, Karin Metzner, Nicolas Müller, Dunja Nicca, Paolo Paioni, Giuseppe Pantaleo, Matthieu Perreau, Andri Rauch, Christoph Rudin, Alexandra Scherrer, Patrick Schmid, Roberto Speck, Marcel Stöckle, Philip Tarr, Alexandra Trkola, Pietro Vernazza, Noémie Wagner, Gilles Wandeler, Rainer Weber, Sabine Yerly

**Affiliations:** 1 School of Life Sciences, EPFL, Lausanne, Switzerland; 2 Swiss Institute of Bioinformatics, Lausanne, Switzerland; 3 Genentech Inc, 1 DNA Way, South San Francisco, CA USA; 4 Department of Genetic Medicine and Development, University of Geneva Medical School, Geneva, Switzerland; 5 JC Wilt Infectious Diseases Research Centre, Public Health Agency of Canada, Winnipeg, MB Canada; 6 Department of Medical Microbiology and Infectious Diseases, University of Manitoba, Winnipeg, MB Canada; 7 Department of Biomedicine, University Hospital Basel, University of Basel, Basel, Switzerland; 8 Division of Infectious Diseases, Regional Hospital Lugano, Lugano, Switzerland; 9 Division of Infectious Diseases, University Hospital Lausanne, University of Lausanne, Lausanne, Switzerland; 10 Division of Infectious Diseases and Hospital Epidemiology, University Hospital Zurich, University of Zurich, Zurich, Switzerland; 11 Division of Infectious Diseases and Hospital Epidemiology, Cantonal Hospital St.Gallen, St.Gallen, Switzerland; 12 Childrens Hospital of Eastern Switzerland, St. Gallen, Switzerland; 13 Department of Infectious Diseases, Bern University Hospital, University of Bern, Bern, Switzerland; 14 Division of Infection and Immunity, University College London, London, UK; 15 Precision Medicine Unit, Lausanne University Hospital and University of Lausanne, Lausanne, Switzerland; 16 Division of Infectious Diseases and Hospital Epidemiology, University Hospital Basel, University of Basel, Basel, Switzerland; 17 Institute of Medical Virology, University of Zürich, Zurich, Switzerland; 18 Basel Institute for Clinical Epidemiology and Biostatistics, University Hospital Basel, University of Basel, Basel, Switzerland; 19 Division of Infectious Diseases, University Hospital Geneva, University of Geneva, Geneva, Switzerland; 20 Institute of Microbiology, University Hospital Lausanne, University of Lausanne, Lausanne, Switzerland; 21 Centre for Laboratory Medicine, St. Gallen, Canton St. Gallen Switzerland; 22 Institute of Social and Preventive Medicine, University of Bern, Bern, Switzerland; 23 Clinic for Infectious Diseases and Hospital Hygiene, Kantonsspital Aarau, Aarau, Switzerland; 24 Deputy of the Patient Organization “Positive Council”, Zurich, Switzerland; 25 Division Infection Diagnostics, Department Biomedicine - Petersplatz, University of Basel, Basel, Switzerland; 26 Division of Infectious Diseases and Hospital Epidemiology, University Hospital Basel, Basel, Switzerland; 27 Clinic for Obstetrics, University Hospital Basel, University of Basel, Basel, Switzerland; 28 Division of Infectious Diseases and Laboratory of Virology, University Hospital Geneva, University of Geneva, Geneva, Switzerland; 29 Institute of Global Health, University of Geneva, Geneva, Switzerland; 30 Ospedale Regionale Di Bellinzona E Valli, Bellinzona, Switzerland; 31 Cantonal Institute of Microbiology, Bellinzona, Switzerland; 32 Department of Obstetrics and Gynecology, University Hospital Geneva, University of Geneva, Geneva, Switzerland; 33 University Children’s Hospital, University of Zurich, Zurich, Switzerland; 34 Division of Immunology and Allergy, University Hospital Lausanne, University of Lausanne, Lausanne, Switzerland; 35 University Childrens Hospital, University of Basel, Basel, Switzerland; 36 Data Centre, Swiss HIV Cohort Study, Zurich, Switzerland; 37 Kantonsspital Baselland, University of Basel, Basel, Switzerland; 38 Pediatria, University Hospital Geneva, University of Geneva, Geneva, Switzerland; 39 Laboratory of Virology, University Hospital Geneva, University of Geneva, Geneva, Switzerland

Correction to: *Scientific Reports* https://doi.org/10.1038/s41598-021-84070-7, Published online 25 February 2021

Nina Khanna, Enos Bernasconi, Matthias Cavassini, Huldrych F. Günthard, Christian R. Kahlert and Andri Rauch were omitted from the author list in the original version of this Article.

The Author contributions section now reads:

"E.Z. and J.F. conceived and supervised the work. S.R. and C.H. performed the association analyses. D.P.D., S.M. and J.B. performed EBV sequencing and curated the data. A.L. prepared and analysed the viral sequencing data. P.J.M., D.L. and O.N. contributed to the design of the work. N.K, E.B., M.C., H.F.G., C.R.K. and A.R. recruited the study participants and collected the samples and associated data. S.R., C.H., A.L. and J.F. wrote the paper. All authors reviewed the manuscript and approved the submission.”

Furthermore, the original version of this Article contained typographical errors in the spelling of Huldrych F. Günthard and Christian R. Kahlert, which were incorrectly given as Huldrych Günthard and Christian Kahlert respectively.

This has been corrected in the PDF and HTML versions of the Article, and in the accompanying Supplementary Information file.

